# Physiological Significance of the Force-Velocity Relation in Skeletal Muscle and Muscle Fibers

**DOI:** 10.3390/ijms20123075

**Published:** 2019-06-24

**Authors:** Haruo Sugi, Tetsuo Ohno

**Affiliations:** 1 Department of Physiology, School of Medicine, Teikyo University, Tokyo 173-8605, Japan; 2 Department of Molecular Physiology, Jikei University School of Medicine, Tokyo 105-8461, Japan; te269ohno@docomo.ne.jp

**Keywords:** *P–V* relation, sliding filament mechanism, skinned muscle fiber, muscle energy output

## Abstract

The relation between the force (load) and the velocity of shortening (*V*) in contracting skeletal muscle is part of a rectangular hyperbola: (*P* + a) *V* = b(*Po* − *P*); where *Po* is the maximum isometric force and a and b are constants. The force–velocity (*P–V*) relation suggests that muscle can regulate its energy output depending on the load imposed on it (Hill, 1938). After the establishment of the sliding filament mechanism (H.E. Huxley and Hanson, 1954), the *P*–*V* relation has been regarded to reflect the cyclic interaction between myosin heads in myosin filaments and the corresponding myosin-binding sites in actin filaments, coupled with ATP hydrolysis (A.F. Huxley, 1957). In single skeletal muscle fibers, however, the *P*–*V* relation deviates from the hyperbola at the high force region, indicating complicated characteristics of the cyclic actin–myosin interaction. To correlate the *P*–*V* relation with kinetics of actin–myosin interaction, skinned muscle fibers have been developed, in which the surface membrane is removed to control chemical and ionic conditions around the 3D lattice of actin and myosin filaments. This article also deals with experimental methods with which the structural instability of skinned fibers can be overcome by applying parabolic decreases in fiber length.

## 1. Introduction

**Figure 1:**
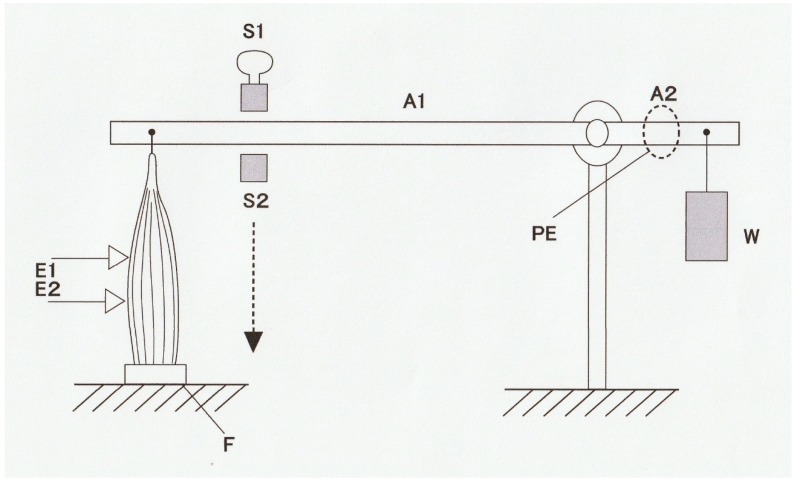
Experimental setup to record the force–velocity relation of skeletal muscle. A frog sartorius muscle (M) is held vertically between the long arm (A1) of the lever and a force transducer (F), while a weight W is put to the short arm (A2) of the lever to apply constant afterload (*P)* to the muscle. Muscle length is initially adjusted by stops (S1 and S2) at a slack length (Lo) where resting force is just barely perceptible. Then, the muscle is stimulated by a single electrical current pulse through a pair of electrodes (E1 and E2) to produce a twitch (at 0 °C). Force and length changes in the muscle during a twitch are recorded by a force transducer (F) and a photoelectric device (PE), respectively.

**Figure 2:**
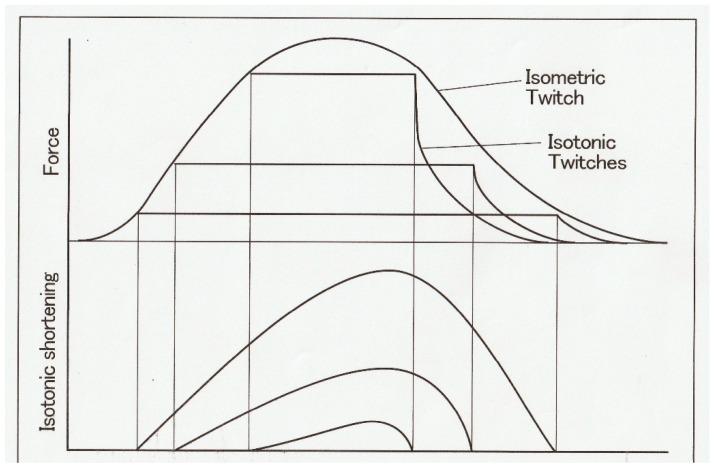
Force (upper traces) and length (lower traces) changes of a muscle during twitches under three different afterloads. The muscle first develops isometric force, and when the force reaches the value equal to the afterload, it starts shortening under the constant afterload with a constant velocity.

Muscle contraction can be characterized by the velocity of shortening and the magnitude of isometric force (tension). Figure 1 shows a diagram of an experimental set-up with which force and shortening velocity can be measured simultaneously. A skeletal muscle (M) is mounted vertically between the long arm of a lever (A1) and the force transducer (F) at its slack length (Lo) with an adjustable stop (S1), while a weight (W) is attached to the short lever arm (A2). Then, the muscle is stimulated electrically with a pair of electrodes (E1 and E2) to produce a twitch (at 0 °C). The muscle first starts generating force without changing its length (isometric force generation). When the magnitude of isometric force reaches the value equal to the amount of load (*P*) imposed on the muscle by the weight (W), the muscle starts shortening with a constant velocity (*V*). If the long arm is sufficiently longer than the short arm, the muscle shortens under a constant afterload (*P*; afterloaded isotonic shortening). Figure 2 shows general features of force and length changes during the afterloaded isotonic twitches under three different afterloads. Afterloaded twitch consists of two phases: the initial phase of force generation from zero to *P* and the subsequent shortening phase under a constant load *P*.

**Figure 3:**
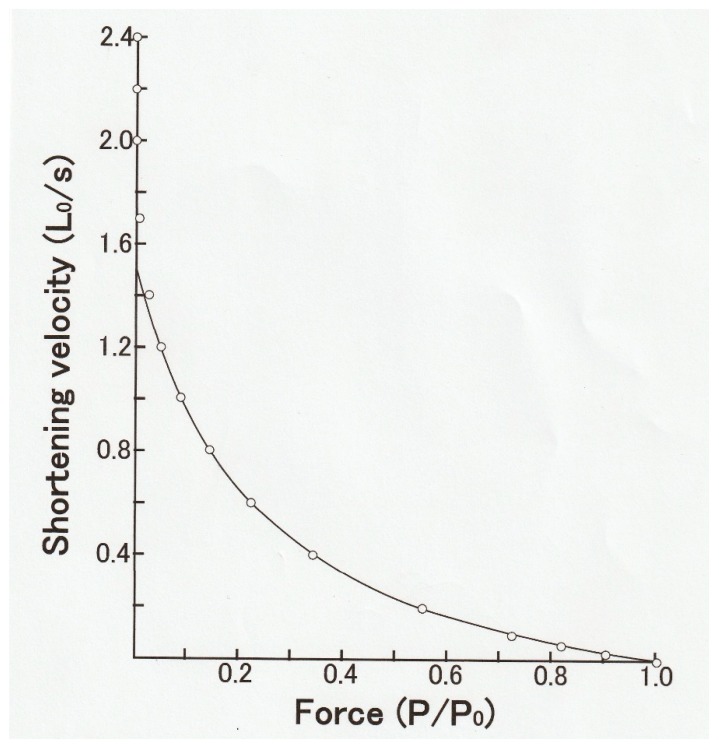
The force–velocity (*P–V*) relation of a whole frog sartorius muscle obtained during an afterloaded twitches at 0 °C. The *P–V* curve fits part of rectangular hyperbola, so that the relation between *P* and *V* are expressed as (*P* + *a*) *V = b* (*Po − P*). From Ref. [1].

In 1938, Hill reported that the shape of the relation between *P* and *V* in frog skeletal muscle (*P–V* relation) was part of rectangular hyperbola, so that (*P* + a) *V* = b(*Po* − *P*), where a and b are constants (Figure 3) [1]. This equation is called the Hill equation and was taken to indicate the energy output rate in a contracting muscle, which consists of the work output (*PV*) and the heat production (a*V*), depending on the amount of external load (*P*). The important values in the Hill equation are (1) the maximum velocity of shortening under zero load (*V*max), the maximum isometric force (*Po*), and a/*Po*, which determines the curvature of the hyperbolic *P–V* relation. The Hill equation is now regarded as a mere empirical equation due to the complex structure of a whole muscle containing different types of muscle fibers, blood vessels, and connective tissues. Nevertheless, many investigators in the field of exercise physiology study the effects of exercise training on the *P–V* relation of skeletal muscle on the basis of the change in a/*Po*.

In this article, we intend to provide a brief overview of studies on the *P–V* relation of isolated single intact and skinned muscle fibers to obtain information about kinetic properties of myosin heads interacting with actin to produce muscle contraction, and also provide a brief description of the results obtained from our electron microscopic work on myosin head power and recovery strokes.

## 2. *P–V* Relation Obtained from Single Intact Muscle Fibers Deviates from the Hyperbolic Hill Equation in the High Force (Load) Region

Although isolation of single muscle fibers from whole muscles requires technical skill, the use of single fibers has the following advantages over the use of whole muscles: (1) A single muscle fiber is a structural and functional unit at the cellular level and is free from complications arising from whole muscles; (2) single fibers isolated without damage survive for many hours to yield reproducible results; (3) sarcomere spacings can be clearly observed under a light microscope, so that length changes of sarcomeres, a structural and functional unit at the subcellular level, can be recorded. *P*properties of attachment–detachment cycle between myosin head in myosin filaments and the corresponding myosin-binding sites in actin filaments. In single fiber experiments, the fibers are first tetanized isometrically with repetitive electrical pulses to produce a steady maximally activated state. At room temperatures, peak twitch force is only a fraction of that attained during isometric tetanus, indicating that, during a twitch, activation of myosin heads is incomplete; at 0 °C, peak isometric twitch force increases but is still lower than peak tetanus force.

**Figure 4:**
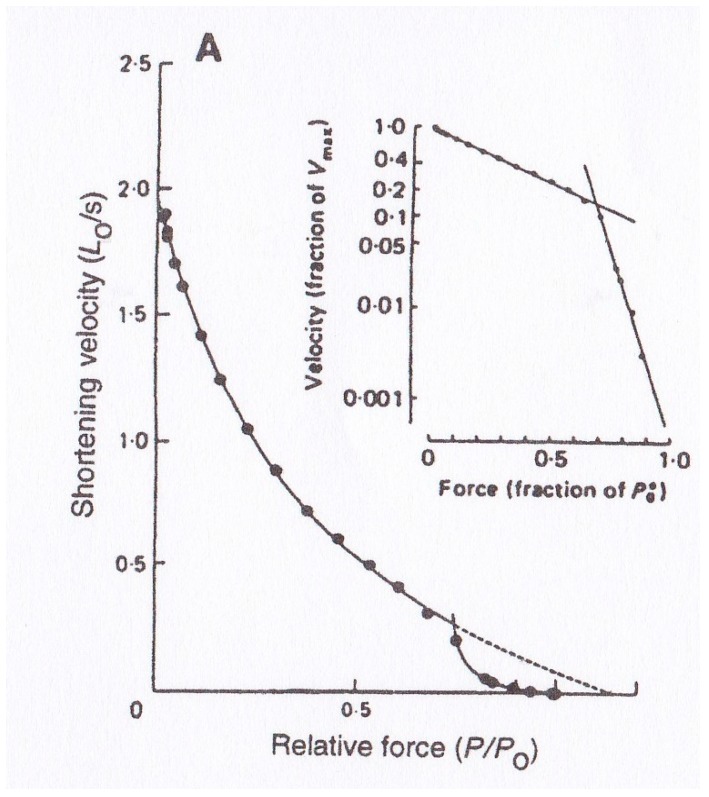
Double-hyperbolic *P–V* relation obtained from single tetanized intact frog muscle fibers. Inset shows semi-logarithmic plot of the same *P–V* data points. From [2].

Edman [2] reported that the *P–V* relation of tetanized intact single skeletal muscle fibers deviated from the Hill equation at high force (load) region, as shown in Figure 4. This finding has been confirmed on intact single fibers [3] and on skinned muscle fibers [4,5,6,7]. The deviation of the *P–V* relation from the Hill equation indicates that kinetic properties of the attachment–detachment cycle between myosin heads and actin filaments are different between low load and high load regions. Though Edman et al. [8] presented a model that explained the deviation of the *P–V* relation from the Hill hyperbolic equation in terms of the properties of the rate constant for formation of the A–M link, its validity cannot be supported experimentally. This issue concerning the double hyperbolic *P-V* relation therefore remains a mystery at the molecular level.

## 3. Studies on the Underlying Mechanism of the Constant Velocity of Isotonic Shortening

**Figure 5:**
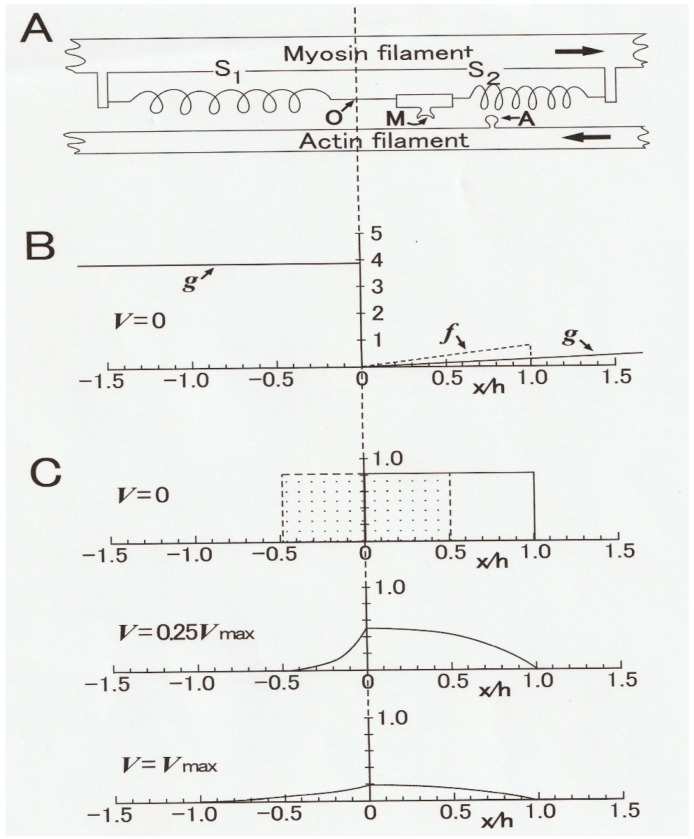
Huxley contraction model, constructed to account for the *P–V* relation of Hill in terms of distribution of the A–M link between myosin head (M) extending from a myosin filament and corresponding myosin-binding site (**A**) in an actin filament. (**A**) Diagram showing a myosin head (M) connected to a myosin filament via springs S1 and S2, and a myosin-binding site (**A**) on an actin filament. Arrows indicate directions of relative sliding between actin and myosin filaments. (**B**) Diagram showing the rate constants (*f* and *g)* as functions of distance (*x*) of M from its equilibrium position (0). (**C**) Diagrams showing distribution of the A–M link along the *x* axis at *V =* 0 (isometric condition), at *V=* 0.25*Vmax*, and *V = Vmax.* For further explanation, see text. From Ref. [9].

In 1957, A.F. Huxley [9] constructed a contraction model (Huxley contraction model), in which mechanisms underlying the constant velocity of isotonic shortening, found by Hill [1], were explained in terms of the steady distribution of myosin heads attached to actin filaments as a function of distance from the myosin head equilibrium position (O). As illustrated in Figure 5A, a myosin head (M) extending from a myosin filament fluctuates around an equilibrium position (O) due to thermal motion (A). The myosin head elasticity is expressed by springs (S1 and S2). In contracting muscle, M attaches to a myosin-binding site (A) of an actin filament to form an A–M link and generates positive force at the right side of O, and negative force at the left side of O. Both positive and negative forces are proportional to the distance *x* of A–M from O.

When M attaches to A at the right side of O (positive *x* region), it generates positive force proportional to the distance *x* from O, while it generates negative force at the left side of O (negative *x* region). The rate constants for making and breaking of an A–M link (*f* and *g,* respectively) are functions of *x* (Figure 5B) and are chosen to fit Hill’s *P–V* relation and also the *P*-energy output relation at that time. In the isometric condition, in which a muscle generates the maximum isometric force *Po* without shortening, A–M links are formed in the region 0 ≤ *x* ≥ *h*, where *f* > *g* (Figure 5C, upper diagram). When the muscle is rapidly released by *h/2,* the rectangular-shaped A–M link distribution shifts to the left, so that the force drops from *Po* to zero at the moment of release as the positive and the negative forces generated by A–M links become equal.

During the shortening with a constant velocity *V* under a constant load (force) *P*, a number of M slide past A from right to left along the *x* axis so that the number of A–M links formed in the positive force region decreases with increasing value of *V,* while the A–M link brought into the negative force region—where *g* is large and *f* = 0 (Figure 5B)—decreases rapidly with increasing *x* from O. The steady A–M link distribution along the *x* axis under a load *P* (= 0.25*Po*) is shown in the middle diagram in Figure 5C. The difference between positive and negative forces at both sides of O is equal to the value of the isotonic load. The lower diagram in Figure 5C shows the steady A–M link distribution along the *x* axis under zero load (*P =* 0) during shortening with the maximum shortening velocity *Vmax.* In this condition, positive and negative forces at both sides of O are just equal.

The Huxley contraction model, described above, stimulated muscle physiologists, and a number of papers have been published in which experimental results were interpreted in the framework of this contraction model. As the result, considerable progress has been made on the kinetic properties of myosin heads interacting with actin filaments, as will be described in the following sections.

## 4. Isotonic Velocity Transients at the Beginning of Isotonic Shortening

**Figure 6:**
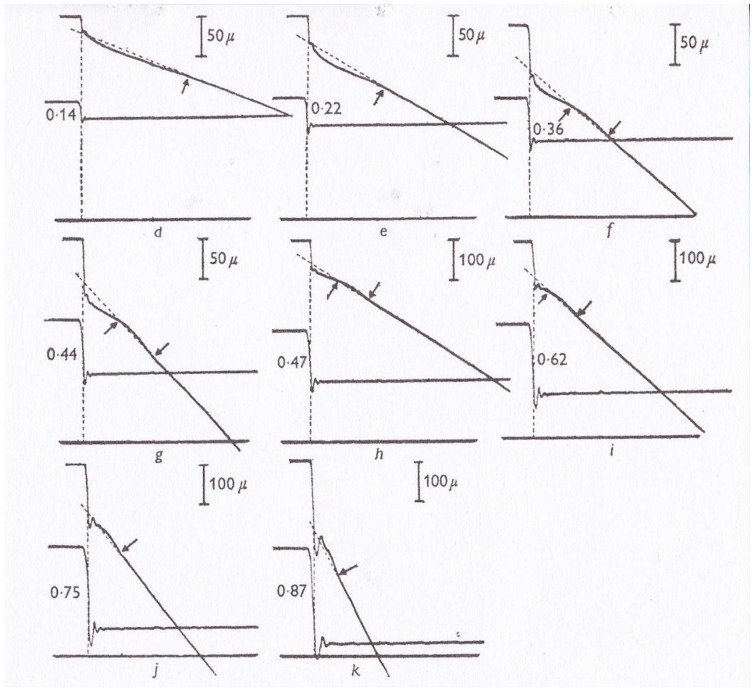
Transient oscillatory changes in shortening velocity (isotonic velocity transient) in tetanized single intact frog muscle fibers following various magnitudes of quick changes in load from *Po* to *P* < *Po.* Changes in fiber length and force are shown in upper and lower records, respectively. From Ref. [10].

In 1966, Civan and Podolsky [10] reported that when a tetanized skeletal muscle fiber generating the maximum isometric force *Po* was subjected to a quick decrease in load (force) from *Po* to *P* < *Po,* non-steady oscillatory changes in shortening velocity preceded the subsequent steady shortening with constant velocity (Figure 6). Since the Huxley contraction model only deals with steady isotonic shortening, Podolsky and Nolan [11] constructed a new contraction model to simulate the initial oscillatory changes in shortening velocity (isotonic velocity transients). Their contraction model is essentially a modification of the Huxley contraction model, assuming a gap in *g* function in the negative *x* region.

**Figure 7:**
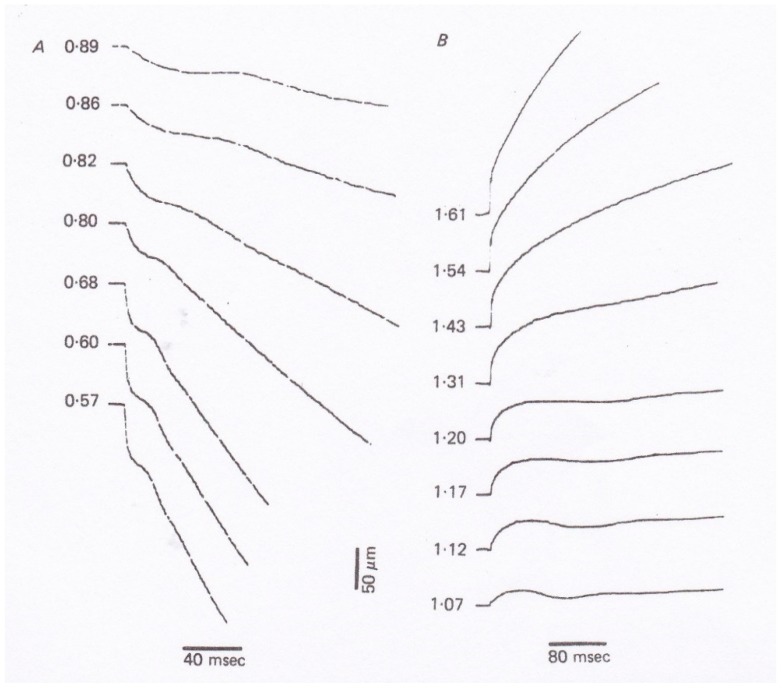
Isotonic velocity transients following quick changes in load from (**A**) *Po* to *P* < *Po* and (**B**) *Po* to *P* > *Po* in tetanized intact single muscle fibers. The values of *P* are expressed relative to *Po* on the left of each record. From Ref. [12].

**Figure 8:**
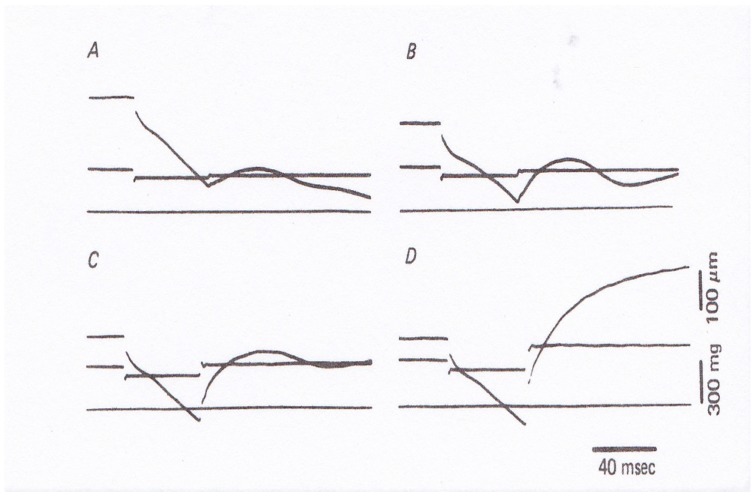
Records of fiber length (upper traces) and force (lower traces) of experiments in which the tetanized intact muscle fiber was first made to shorten isotonically under a load 0.8*Po* and, after 50 ms, subjected to quick increases in load from 0.8 *Po* to 0.85*Po* in (**A**)*,* to 0.9*Po* in (**B**), to 1.1*Po* in (**C**), and to 1.3*Po* in (**D**). Note marked changes in fiber length with alternate lengthening and shortening in (**A**–**C**). From Ref. [12].

In 1981, Sugi and Tsuchiya [12] applied rapid force changes to isometrically tetanized intact muscle fibers not only from *Po* to *P* < *Po* but also from *Po* to *P* > *Po* (Figure 7), and they found that (1) the velocity of isotonic lengthening was not constant but decreased with time or with distance of lengthening; and (2) following small step increases in load from *Po* to *P* < 1.2*Po,* the fiber exhibited distinct oscillatory length changes consisting of alternate lengthening and shortening (Figure 7B and Figure 8). They also constructed a new contraction model to explain the distinct initial fiber length changes [12]. Although the experiments to apply quick changes in load to isometrically contracting fibers give interesting results on kinetic properties of myosin heads interacting with actin filaments, it was not possible to obtain definite information about the mechanism of contraction at the molecular level; in other words, it was not possible up to the present time to explain how the rate constants *f* and *g* in the contraction models actually correspond to the three-dimensional structures of myosin heads in myosin filaments and actin monomers in actin filaments.

## 5. Constant Velocity Isotonic Shortening in an Afterloaded Twitch in Single Skeletal Muscle Fibers without Preceding Velocity Transients

**Figure 9:**
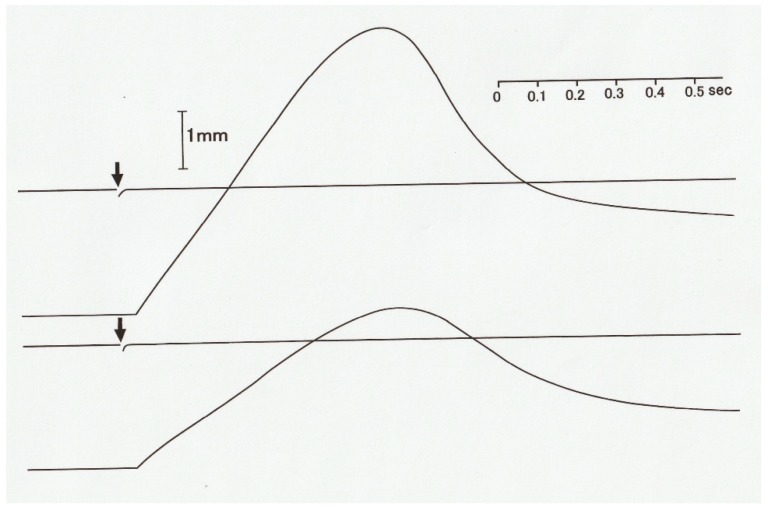
Records of isotonic shortening of an intact single muscle fiber during afterloaded twitches under two different afterloads. Note that a single fiber starts shortening linearly after preceding isometric force development equal to the afterload without any transient changes. From Ref. [13].

In contrast with the distinct velocity transients observed in maximally tetanized intact muscle fibers, preceding the steady isotonic shortening [10,12], the constant velocity isotonic shortening without preceding velocity transients has been observed in single muscle fibers during an afterloaded twitch at 0 °C (Figure 9) [13]. This suggests that the state of myosin heads during the generation of steady isomeric force *Po* may be definitely different from that of myosin heads during the generation of submaximal force preceding isotonic shortening in an afterloaded twitch. It may be that, due to definite difference between the “steady isometric force generation mode” and the “steady isotonic shortening mode” of myosin head operation, myosin heads should pass through transient changes in their state of operation, which may show up as isotonic velocity transients [10,12].

On the other hand, during an afterloaded twitch, the steady mode of isometric force generation of myosin heads is not yet established, and consequently, muscle fibers start shortening under a constant load without being preceded by oscillatory changes in shortening velocity [13], as has been the case in whole muscles during an afterloaded twitch [1]. This implies that during the initial force development preceding shortening, the mode of operation of myosin heads is nearly similar to that during shortening, since myosin heads should shorten internally by stretching the series elastic component [14].

**Figure 10:**
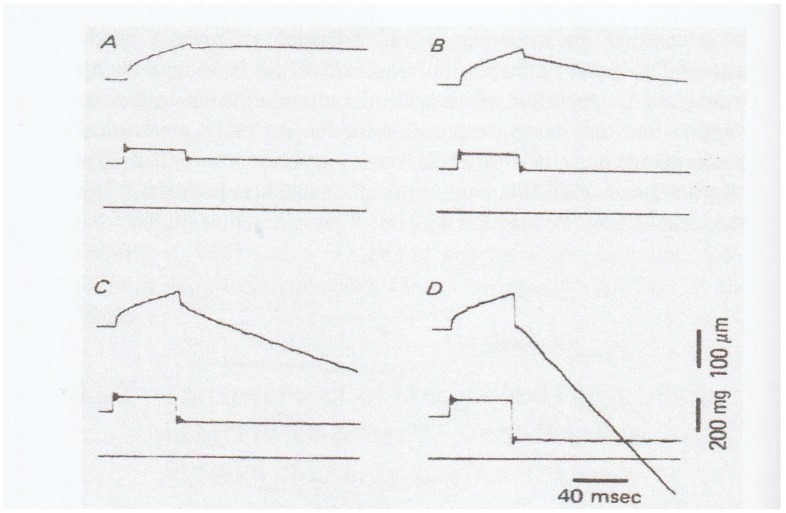
Enhancement of mechanical performance in tetanized intact muscle fibers when they are first lengthened by quick changes in load from *Po* to *P* > *Po* and then subjected to quick changes in load from *P* > *Po* to *P < Po*. Note that the fiber shortens under a load = *Po* (**B**), as well as under loads *P* < *Po* (**C**, **D**), while it is lengthened under a load > Po (A); and also note that the fiber starts shortening without any velocity transients. From Ref. [15].

**Figure 11:**
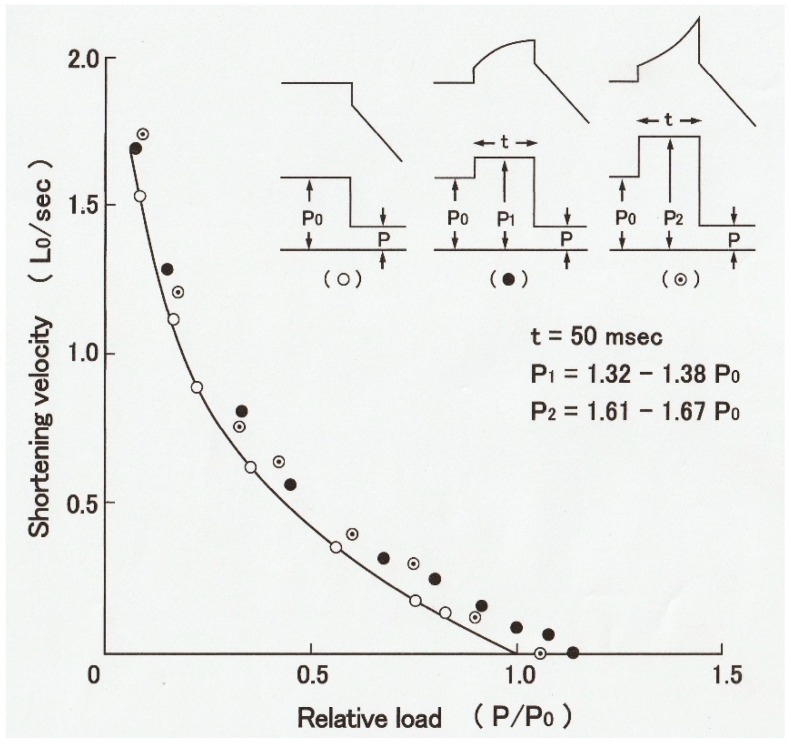
Shift of the *P–V* relation to the right along the force (load) axis as the result of enhancement of mechanical performance in isotonically lengthening muscle fibers. Open circles and the solid line show the *P–V* relation, obtained in the control condition, while filled and dotted circles show data points, obtained during isotonic lengthening under a load of 1.32~1.38*Po* and 1.61~1.67*Po*, respectively. Note that the shift of the *P–V* curve occurs at all levels of shortening velocity. From Ref. [15].

In this connection, it is of interest that when muscle fibers generating steady isometric force are first lengthened isotonically under a load *P1* > *Po* and then subjected to a quick decrease in load from *P1* to *P2* < *Po,* they start shortening with a constant velocity without showing oscillatory velocity transients (Figure 10) [15]. This result implies that the mode of myosin head operation may be nearly similar to each other during both isotonic shortening and isotonic lengthening, so that no transient velocity changes are observed when isotonically lengthening fibers are suddenly made to shorten isotonically. It should also be noted that the mechanical performance of muscle fibers is enhanced after isotonic lengthening. As can be seen in Figure 10B, the fibers can shorten under a load equal to the maximum isometric force *Po.* The enhancement of mechanical performance in isotonically lengthening fibers can be seen over the whole range of load to result in the shift of the *P–V* curve to the right along the force (load) axis (Figure 11B).

## 6. Electron Microscopic Visualization and Recording of ATP-Induced Myosin Head Movement in Hydrated, Living Myosin Filaments

As stated above, the *P–V* relation of muscle fibers gives information about the mode of operation of myosin heads interacting with actin filaments. Despite extensive studies on contracting muscle by the methods of chemical probes attached to myosin heads [16] and time-resolved X-ray diffraction [17], it has not yet been possible to clearly characterize myosin head states during power and recovery strokes, mainly due to the asynchronous nature of myosin head movement. Up to the present time, one of the most effective experimental means to investigate the mode of operation of individual myosin heads under electron microscope is the use of a gas environmental chamber (EC), which enables us to visualize and record ATP-induced myosin head movement in hydrated, living myosin filaments [18,19]. The EC method is based on position-marking of individual myosin heads in hydrated myosin filaments by attaching gold particles to different regions within myosin head via different antibodies directed against the myosin head, and it has the following advantages over static crystallographic studies of myosin heads: (1) It can record the amplitude of individual myosin movement in hydrated myosin filaments, which retain their physiological function; and (2) it can record the amplitude of myosin head movement at three different regions within a myosin head so that information about changes in myosin head configuration during its power and recovery strokes can be obtained.

**Figure 12:**
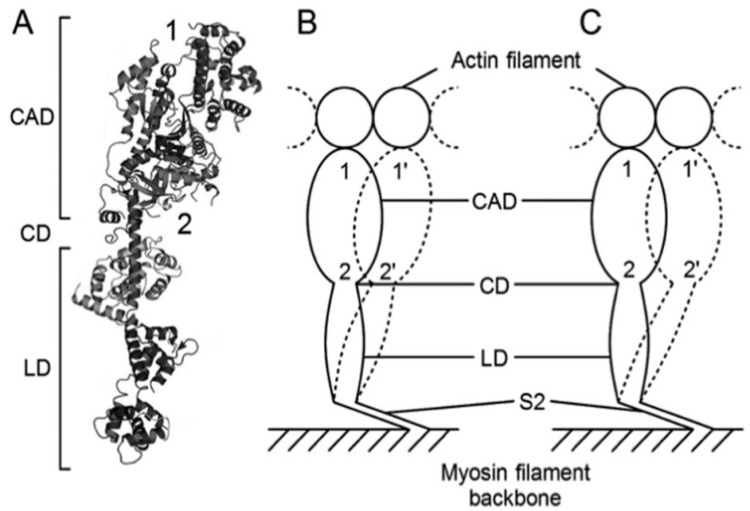
Two different modes of ATP-induced myosin head power strokes. (**A**) Structure of the myosin head, which consists of a catalytic domain (CAD), a converter domain (CD), and a lever arm domain (LD). Approximate attachment regions of antibodies 1 and 2 are indicated by numbers 1 and 2, respectively. (**B**) The mode of myosin head power stroke at standard ionic strength. The amplitude of movement is larger at the distal CAD than at the proximal CAD so that the myosin head is oblique to actin and myosin filaments at the end of a power stroke. (**C**) The mode of a myosin head power stroke at low ionic strength. The amplitude of movement is the same at both the distal and the proximal CAD so that CAD is perpendicular to actin and myosin filaments at the end of power stroke. From Ref. [19].

Since the results and their implications by the EC method have been described in detail elsewhere [20,21], we will only describe our recent results on the two different modes of the myosin head power stroke in the actin-myosin filament mixture [19], where individual myosin heads are position-marked with two different antibodies: antibody 1, attaching to the distal region of the catalytic domain (CAD), and antibody 2, attaching to the proximal region of CAD (Figure 12A) [22]. In the EC experiment, only a small fraction of myosin heads can be activated with iontophoretically applied ATP so that individual myosin heads, activated by ATP, cannot cause gross myofilament sliding but only stretch adjacent elastic structures during their power stroke. In the standard ionic strength (µ = 170 mM), the average amplitude of the power stroke is ~3.3 µm at the distal region of CAD and ~2.4 µm at the proximal region of CAD. As a result, myosin head CAD is oblique to actin filaments at the end of a power stroke (Figure 12B). At low ionic strength (µ = 50 mM), which is known to enhance isometric force generation of skinned muscle fibers two-fold without changing ATPase activity [23], the average amplitude of a power stroke in the CAD increases to ~4.5 µm at both distal and proximal regions so that the CAD is perpendicular to actin filaments at the end of power stroke at present (Figure 12C). Therefore, the EC method can provide information about mechanism underlying the changes of *P–V* relation by recording myosin head positions at both the distal and the proximal myosin head CAD before and after power and recovery strokes. At present, the shortcomings of the EC method is its limited time resolution (0.1 s), which should be improved in the future.

## 7. Studies on the Mechanism Underlying Muscle Fatigue by Recording *P–V* Relation: Problems Arising from the Use of Skinned Muscle Fibers

It has long been known that after a long-lasting contractile activity, the ability of skeletal muscle to generate force and power decreases due to fatigue [24], which is believed to result from products of ATP hydrolysis (Fitts, 1994) [25]. To study the effect of increased inorganic phosphate (Pi) as well as pH in muscle fibers, skinned muscle fibers are used, in which surface membrane is removed mechanically or chemically so that ionic and chemical conditions around myofilaments can be controlled.

Experiments with skinned fibers have, however, inherent difficulties in obtaining reproducible results over a long period of time, because they slowly deteriorate at their cut ends [26]. The most important parameters in the *P–V* relation are a/*Po* and *Vmax,* i.e., the maximum shortening velocity at zero external load. The value of *Vmax* is generally believed to give information about the maximum cycling rate of actin–myosin interaction in muscle fibers. As it is technically difficult to make muscle fibers shorten isotonically under zero external load, the value of *Vmax* can only be roughly estimated by back-extrapolation of data points to the velocity axis (Cooke & Pate, 1985; Cooke et al., 1988) [27,28]. In intact muscle fibers, *Vmax* can be obtained more accurately by the slack test (Edman, 1979) [29], in which *Vmax* is obtained from the time of force redevelopment after the fiber is slackened excessively. Unfortunately, the slack test cannot be used successfully for skinned fibers because of deteriorating fiber ends.

**Figure 13:**
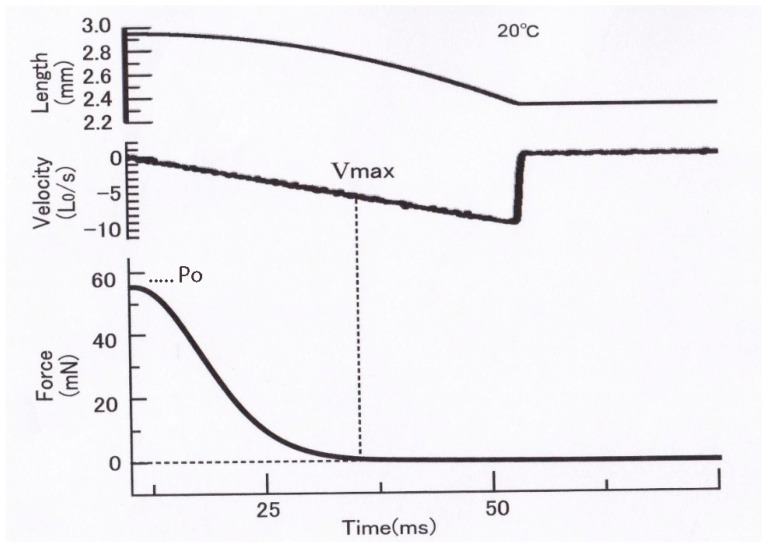
Recording of the whole range of *P–V* relation in one shot by applying a slow parabolic decrease in length of a single skinned muscle fiber, generating the maximum Ca^2+^-activated isometric force. (Upper record) Parabolic decrease in fiber length (*L*), *L = −kt^2^*. (Middle record) Linear velocity of fiber shortening (*V*), *V = −dL/dt = −2k.* (Lower record) Time course of the resulting decrease of force (*P*) in the fiber from *Po* to 0 along the vertical force axis. Note that *V =* 0 when *P = Po,* and *V = Vmax* when *P =* 0, so that the horizontal time *t* axis serves as velocity *V* axis. Therefore, the lower force record per se constitutes the *P–V* relation over the whole range of *P* from 0 to *Po*. From Ref. [7].

Recently, we have developed a novel method to obtain the *P–V* relation in single skinned skeletal muscle fibers over the whole continuous range of loads from zero to *Po* in one shot by applying parabolic length changes to Ca^2+^-activated rabbit psoas muscle fibers, and we succeeded in obtaining reproducible *P–V* relations without deterioration of the fiber due to repeated application of isotonic shortening under various loads [7]. As illustrated in Figure 13, a single skinned muscle fiber was first maximally activated with contracting solution (pCa, 4) to generate isometric force *Po* and then subjected to a slow decrease in fiber length (*L*) (duration: 50–500 ms, upper record). The time course of the slow decrease in *L* was part of the upside-down parabola so that *L* decreased with the square of time, as *L =* −*kt^2^*, where *k* is a constant. The velocity of decrease in fiber length *V* at time *t* is therefore, *V = dL/dt = −2kt*; namely, the value of *V* increases linearly with time *t*. Therefore, the velocity of fiber shortening (*V*) increases linearly with time (middle record) along the horizontal time axis. As a consequence, the force in the fiber (*P*) decreased from *Po* to zero (lower trace) along the vertical force axis (lower record). As shown by the dotted vertical and horizontal lines, *V =* 0 when *P = Po*, and *V = Vmax* when *P =* 0; the force record per se constitutes the *P–V* relation over the whole force range from *Po* to zero. Thus, it was possible to obtain *P–V* relations in skinned muscle fibers over the whole range of isotonic load (force) in one shot, including *Vmax*, which is difficult to determine accurately by the slack test.

**Figure 14:**
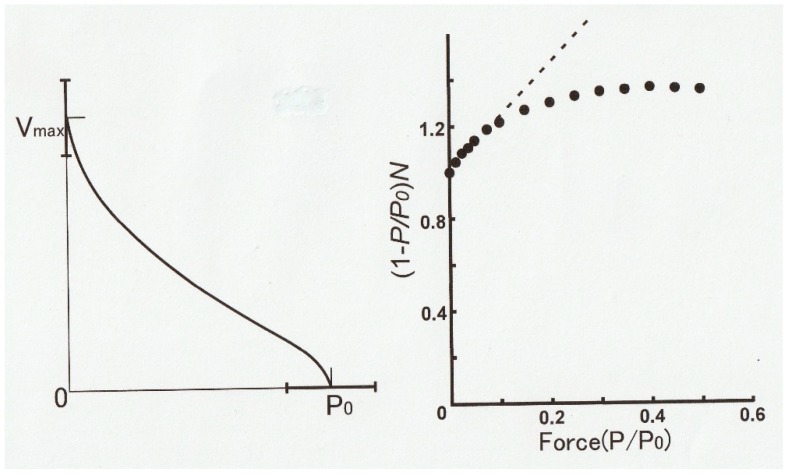
(Left) Averaged *P–V* relation of skinned muscle fibers obtained from six different fibers. Horizontal bars show SD. (Right) Linearized Hill equation plot of data points selected from the above *P–V* curve. The quantity, (1 − *P/Po*)*V* (filled circles), is plotted against relative force, *P /Po.* If the *P–V* curve in A fits the Hill equation [1], the data points should fall on dotted straight line. The data points fit Hill equation only forces <~0.1*Po*. From Ref. [30].

We studied the effect of inorganic phosphate Pi and low pH on the *Vmax* and *Po* to give information about mechanisms underlying muscle fatigue, all without complications arising from deteriorating cut ends of skinned fibers. Figure 14 (left) is an example of a *P–V* relation obtained in one shot by the method illustrated in Figure 13. The *P–V* relation thus obtained not only exhibits distinct hump in the high load region but also markedly deviates from the hyperbola, i.e., the Hill equation [1]. Figure 14 (right) shows a linearized Hill equation plot (dotted straight line) where the quantity (1 − *P/Po*)/*V* is plotted against relative force *P/Po*. The values of *P* and *V* are taken from the *P–V* relation in Figure 2 [1]. If the *P–V* curve obtained by us fits the Hill equation, all the data points (filled circles) should fall on the dotted straight line.

As can be seen in Figure 14 (right), only the data points <~0.1*Po* fall on the dotted straight line, indicating a marked deviation from the Hill equation [1]. A similar marked deviation from the Hill equation has also been reported by Julian [28], who reported on the *P–V* relation in intact single muscle fibers, obtained using length and force clamp techniques.

By the method illustrated in Figure 13, we obtained the following results (at 20 °C): (1) Elevated Pi from zero to 30 mM decreased *Po* by ~25% but had no significant effect on *Vmax*; and (2) reduction of pH from 7.0 to 6.5 decreased both *Po* and *Vmax* by ~20% and ~10%, respectively. The reduction of *Po* by high Pi agrees with previous reports [30,31,32,33] and can be explained to be due to product inhibition of the Pi-releasing step in the actomyosin ATPase reaction preceding force generation [34]. On the other hand, the inhibitory effect of low pH on *Po* cannot be readily explained. Based on in vitro motility experiments, it is suggested that low pH might modify the rate of breaking of the A–M link [35]. As pointed out by Sugi et al. [6], however, in vitro motility assay conditions differ too far from that in contracting muscle. The ineffectiveness of high [Pi] on *Vmax* in our study agrees with the report from Cooke et al. [36]. These results are consistent with the Huxley contraction model [9] where the value of *Vmax* is independent of degree of activation, i.e., the change in the number of myosin heads involved in contraction. However, this explanation seems to contradict the concept of product inhibition of the Pi-releasing step in cyclic action myosin interaction, since *Vmax* is generally believed to reflect the turnover rate of actin–myosin interaction in contracting muscle. More experimental work is necessary to clarify mechanisms underlying the *P–V* relation.

## 8. Conclusions

In this article, we first explained the early work by Hill on the relation between the load and the velocity of shortening (*P–V* relation) during afterloaded twitch in the whole frog skeletal muscle. The *P–V* relation was fitted to part of rectangular hyperbola as (*P* + a)*V =* b(*Po − P*) and was taken to indicate that the rate of energy flux (*PV* + a*V*) is dependent on the load. The Huxley contraction model was constructed to account for the Hill equation. In the field of exercise physiology studying the performance of whole muscle, the value of a/*Po* is widely used to evaluate muscle efficiency. At the level of isolated single muscle fibers, the *P–V* relation was found to deviate from the hyperbola in the high load region in both intact and skinned fibers. Thus, muscle physiologists focused their attention towards the transient phenomena, which arose following transitions from the isomeric force-generating state to the state of isotonic shortening. Despite the considerable progress of research work in this field, it was not possible to prove the validity of the proposed mechanisms of myosin head performance.

In this connection, we emphasize that our electron microscopic recording of ATP-induced position-marked myosin head movement in hydrated, living myosin filaments, mounted in the EC, is the only effective means in connecting knowledge of muscle physiology and those of muscle ultrastructure. It seems possible that a breakthrough will be achieved in the field of muscle research if our EC method is coupled with the method of time-resolved electron microscopy [37].
